# Advancing community health measurement, policy, and practice

**DOI:** 10.7189/jogh.11.01001

**Published:** 2021-03-10

**Authors:** Ben Bellows, Charlotte E Warren

**Affiliations:** 1 Nivi, Inc., Sudbury, Massachusetts, USA; 2 Population Council, Washington DC, USA

Universal access to high quality care is at the core of the Universal Health Coverage (UHC) movement and is foundational for achieving several of the Sustainable Development Goals. Notions of universality in health require a focus on community health, which has received significant recognition and redoubled effort since the 40^th^ anniversary of the Alma Ata Declaration on Primary Health Care (PHC) with the Declaration of Astana in 2018 [1-3]. That same year, the World Health Organization released the Community Health Guideline to accelerate policy reforms and community health program formulation, maturation, and scale in the lived context of each country [4].

Scaling, maintaining, and maturing community health requires overcoming a number of challenges: fragmented funding; weak coordination; disease-specific training; donor-influenced management, funding, and priorities; weak supply and referral linkages with the health system; limited supervision and support, and limited financial and non-financial recognition and remuneration to community health workers (CHWs) [1]. To address these challenges and manage scale-up of community health, insight into programmatic function (eg, measures and data) is needed. Despite 40 years of research and program advocacy, until recently, there was no organizing framework to understand performance metrics for community health and where there may be gaps.

## Importance of performance measures

**Figure 1:**
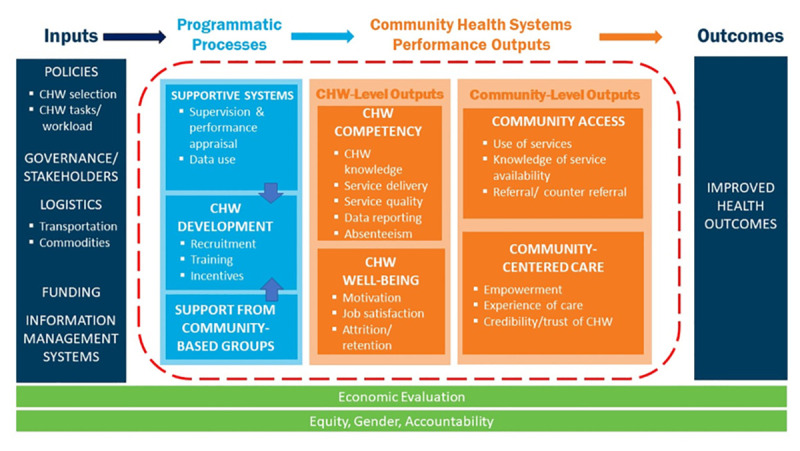
Community Health Worker Performance Measurement Framework, as published in [3].

As part of the Frontline Health (FLH) project in 2019, the Population Council and partners developed a Community Health Worker Performance Measurement Framework that identified 46 service-agnostic indicators across seven performance domains [3]. The framework was developed through an iterative process of literature review and multiple stakeholder consultations to identify the relevant measurement domains of community health performance. Arranged in a common “input -> process -> output -> outcome” logic model (see **Figure 1**), the review identified the following domains and sub-domains within the process and output stages: supportive supervision (supervision and performance appraisal, data use), CHW development (recruitment, training, and incentives), support from community based groups, CHW competency (CHW knowledge, service delivery, service quality, data reporting, absenteeism), CHW well-being (motivation, job satisfaction, attrition/ retention), community access (service use, knowledge of service availability, referral / counter referral), and community-centered care (empowerment, experience of care, credibility / trust of CHWs).

## Supplement extends framework and addresses research gaps

This supplement builds on the FLH framework, exploring qualitatively and quantitatively several of the FLH measurement domains through contextually relevant operational research. The supplement draws on previously identified research gaps related to policy and practice [5] and the WHO recommendation for diverse methodologies to propel the next phase of more complex and maturing community health initiatives [6]. The supplement presents the latest contribution to the measurement and implementation research literature with new studies of measure validation and reliability as well as an expanded understanding of the context of performance metrics in community health programs.

## INPUTS

In the “inputs” phase of the common logic model in **Figure 1**, the contextually relevant inputs include governance, policies, and resources (eg, logistics, funding, and information) that precede the processes and outputs reflected in the framework indicators. In a commentary in this supplement, Leydon et al note the significant shifts in country-led reform in policies and systems towards improving equitable and high-quality community level health care [7]. They identify three priorities to strengthen community in PHC: investments in national priority-setting, service quality, and governance. In a qualitative study in Sierra Leone, Pente and colleagues underscore that a robust and supportive health policy environment is a critical prerequisite if task-shifting is to realize the potential to improve service availability and provision in under-resourced specialities, like ophthalmology [8].

Following a government-led assessment that aimed to nurture a conducive policy environment for community health in Liberia, Simen-Kapeu and colleagues note the importance of five key policy-relevant processes: 1) ensuring government leadership and ownership; 2) establishing a coordination mechanism and leveraging partnership support; 3) conducting evidence-informed planning to inform policy-makers; 4) using a systems approach through a participatory process to better inform policy shifts; and 5) strengthening community engagement and participation in the policy-making process [9].

## SUPPORTIVE SYSTEMS

The first domain within the FLH framework, “supportive systems,” includes performance metrics for supervision and performance appraisal, and data use for decision making within health systems. The FLH framework recommends nine performance indicators.

In this supplement, Sripad and colleagues qualitatively document in Haiti the ability of the community health system (CHS), eg, CHWs, CHW supervisors, and community health auxiliary nurses, to maintain PHC during protracted crises. In their policy-level narrative, the authors emphasize the importance not only of CHWs, but also CHW supervisors, in maintaining a resilient CHS. The performance metrics described by Agarwal et al (2019), eg, CHW-to-supervisor ratios, percentage of CHWs who received a supervisory visit in the previous month, etc., could be the means to monitor the performance of the supervisor-CHW pair and other critical dimensions of Haiti’s resilient CHS. The authors take the notion of CHS resilience further, suggesting it ought to be explored as an adaptive supportive system in other complex humanitarian settings as well as within chronically weak or fragile systems [10].

In a mixed methods operations research study of CHWs’ adaptation to tablet data entry and reporting in Mali, Kirk and colleagues found that while CHW use of tablets is quickly feasible at a small scale and desirable at a large scale, to institutionalize CHW use of community health data requires integration with community health workflows, consistent feedback on skills, and technological support from the administrative center [11]. In short, the challenge to use data once it is collected is encompassed in a larger challenge of changing default data use practices and data literacy within both the community and the health system.

## CHW DEVELOPMENT

Within the FLH framework, the second domain “CHW development” covers performance metrics related to CHW recruitment (eg, number of CHWs recruited), training (eg, percentage of CHWs completing a certificate program) and incentives (eg, number of CHWs who received their monthly stipend on time). The FLH framework recommends eight performance indicators within this domain. For this supplement, one article explored incentives as a sub-domain. In a study of CHW preferences in Uganda, Agarwal and colleagues identify common financial and non-financial incentives and then rank order CHW and policymaker preferences via a discrete choice experiment to identify realistic, pragmatic incentives to improve CHW working conditions with the aim of improving retention. The authors find that non-monetary incentives (eg, identification, transportation) are crucial motivators for CHWs and should be considered as part of the compensation package to facilitate improved performance of CHW programs [12].

## CHW COMPETENCY

Within the FLH framework “CHW competency” domain, performance metrics are linked to sub-domains about CHW knowledge, quantity and quality of services offered, data reporting, and absenteeism. The FLH framework recommends 12 performance metrics within this domain.

In this supplement, three new studies offer insights into measurement of various aspects of CHW competency.

Nested within a larger study in Kenya, Zieman and colleagues identify gaps in the technical quality of CHW postnatal care (PNC) practices, while also recognizing positive elements of experiential quality of care, including communication quality and trusting relationships [13]. Low PNC quality was attributable to a weak supply chain. From a management perspective, the study points to how CHW competence in PNC could be understood as a signal indicator for supply chain performance. The mixed methods study also demonstrates the unique positions that CHWs occupy between the community and facilities, to the extent that the CHWs are perceived as, and empowered to be, an integral part of the PHC network in Kenya.

In Bangladesh, Hossain and colleagues tested the practicality of using the method information index plus (MII+), a measure recently validated elsewhere at the facility level [14,15] to score the quality of counseling in family planning (FP) sessions at the community level [16]. The researchers found that as a measure of FP service quality, the MII+ works well as a means to track CHW competency and service quality.

## CHW WELL-BEING

Within the FLH framework “CHW well-being” domain, performance metrics are linked to sub-domains about CHW motivation, job satisfaction, and attrition/retention. Within the FLH framework, the four recommended performance metrics within this domain were arguably some of the least developed metrics when the framework was published.

Gottert and colleagues validate a multi-dimensional scale to assess CHW motivation, finding it is both a valid and reliable measure comprehensively assessing motivation, orthogonal to specific services [17]. The authors recommend the scale be employed in future research around CHW performance and CHS strengthening worldwide.

## COMMUNITY-CENTERED CARE

Within the FLH framework “community-centered care” domain, performance metrics are linked to sub-domains about trust of CHWs, experience of care, and empowerment. The supplement presents and recommends four composite measures within this domain that were developed and validated across multiple countries using mixed methodologies.

Sripad and colleagues validated a 10-item “trust in CHW” scale in Haiti, Kenya, and Bangladesh, finding it is a reliable and valid tool for quantifying clients’ trust in CHWs, with potential utility for tracking and improving CHW and health systems performance over time [18].

McClair and colleagues developed and validated a client empowerment scale across the same three countries, finding it a valid and reliable construct and associated with greater trust of CHW, satisfaction with CHW services, civic engagement (new scale), and education [19]. A short scale on CHW influence on client empowerment is also recommended for routine monitoring.

In their Kenya PNC study, Zieman and colleagues measured positive elements of respondents’ experience of care, including information recall and the level of trust in the patient-provider relationship [13]. CHWs occupy unique positions as health system agents fostering a positive relationship with community members and supporting PHC in Kenya.

Choudhary recounts experience in building trust in CHWs during polio vaccination campaigns in India, leveraging the social mobilization network. The study finds the social mobilization network to be an effective model of community engagement, and may be useful to achieve the desired outcomes of community health programs elsewhere [20].

## CONCLUSION

Our supplement underscores that implementation research is needed to continue the development, both validation and reliability testing, of performance measures in community health to strengthen performance management. These manuscripts represent our improved collective ability to understand, monitor, manage, and mature community health in diverse socio-political, economic, digital, and geographical contexts.

While continued validation and replication of certain metrics in other community health settings is an ongoing process, the state of measurement has substantially improved over the past few years in alignment with global priorities agendas [6,21]. FLH’s Community Health Worker Performance Measurement Framework enabled synthesizing the state of knowledge and identifying gaps in performance monitoring as well as better managing programs in the future. This framework, further strengthened by the supplement content, emphasizes the importance of community-integrated PHC as a key component of achieving UHC.
